# Annotations

**Published:** 1893-09-02

**Authors:** 


### ANNOTATIONS.

**Parents and Schoolboys' Loyalty.** It is rather a portentous phenomenon that a considerable number of British parents have quarrelled with the authorities of our public schools because they have extended the midsummer vacation for a whole week by way of giving special significance to the marriage-year of the heir presumptive to the Throne of these realms. That loyalty to the Throne is largely sentimental we readily admit, but it is also quite as largely practical. The Throne in this country represents a threefold majesty—the majesty of the Sovereign, the majesty of the law, and the majesty of the people. The instructed Englishman is loyal to this threefold majesty. He sees in it his safety, and the preservation of the greatness and permanence of his country. We are accustomed to regard the class from which public schoolboys are drawn as the most loyal because the most instructed and intelligent of our people. It comes upon us, therefore, with a shock of surprise when we see that class, in large numbers, displaying the temper of Shylock, and insisting upon their full "pound of flesh." Does it mean that loyalty, and poetry, and all the generous qualities which distinguished Shakespeare's Englishmen are dead, and that the "spirit of the counter," so large a parcel of goods for so much money, is the ruling spirit in every home? It would seem so. If we were to argue the question in this spirit, we would say that in these days of "stuffing and cram" an extra week's holiday is an excellent thing, and that, especially having regard to the intensely hot summer, it is even a necessary thing. Boys must have a few weeks of comparative coolness wherein to recover from the heat-languor of the past months, otherwise they are likely to break down before half the new term is over. But we refuse to argue the question on such miserably utilitarian grounds. Britain is still Britain. Her sons still love freedom and loyalty, and a large and easy generosity of mind. If mere money and money's worth are in future to be our only standards of practical life and conduct, then let us put up our national shutters and retire into private and ignominious life. In that case England will be no longer England, and the sooner we write her epitaph the better. Let this be it: "Here lies a country which was great; but falling upon evil times she became mean, and died the death of a dog."

**A Nation of Tea-Drinkers.** WE are a nation of tea-drinkers; we consume about five and a half pounds of the leaf per head annually, which, when made into a beverage, produces about thirty-seven gallons of tea. The question is beginning to arise—Are we a nation of tea-drunkards? For not only are we yielding with all the weakness of the inebriate to the diseases of nerve and stomach which excessive tea-drinking brings in its train, but we are developing that indifference to quality which is the crowning mark of indulgence, the point of severance between the gourmand and the connoisseur. Sir Andrew Clarke's condemnation of Indian teas created a brief reaction in favour of the Chinese leaf, but it did not last long. China tea proved too delicate in flavour for palates vitiated by the coarser product of Assam, and tea-dealers say that after a week or two the demand died away, and consumers returned to their old familiar blends. Tea has always been popular in England, even when its price was enormously high, and when a moralist condemned its consumption as a "filthy custom," to be explained only by the growing wickedness of the nations. This gentleman, Mr. Henry Savile, writing to a friend, speaks with indignation of those who "call for tea, instead of pipes and bottles after dinner, a base unworthy Indian practice, and which I must ever admire your most Christian family for not admitting." What would this old-fashioned Christian of 1678 say to our modern temperance societies and their endless tea drinking? But, indeed, it almost seems as if a new temperance would have to arise to lead a crusade against our favourite beverage, and reformers should petition Parliament to increase the duty on tea. By far the larger part of the tea we drink now is the product of India and Ceylon. From a pound of Indian tea you can make 7½ gallons of infusion; from a pound of Chinese tea only 5 gallons. This consideration is likely to weigh with the average housekeeper, who appreciates an immediate effect on her purse more than a remote effect on the digestions of her household. The result is that nearly 75 per cent. of our tea is of Indian and Cingalese growth. These teas are, moreover, cheaper than the China leaf, and as they are thus doubly tempting, they have attained a dangerous popularity. We drink more tea than our parents; we take it oftener, stronger, and of coarser quality. The results are less obvious than those of alcoholic intoxication, but not less serious; and in truth the time may not be far distant when the earnest disciples of the new temperance will plead with us with tears in their eyes: "Give up this accursed tea, and take to cocoa, or even to beer."

**Leprosy in Trinidad.** A WRITER in *The Illustrated American* describes with a feeling of horror, which he cannot suppress, the leper settlement at Trinidad. The asylum there contains more than two hundred occupants, all, apparently, of African descent. They seem to be, apart even from their disease, a peculiarly unattractive people. Patience is the last virtue they think of. Their quick negro blood, hot by nature and fevered by disease, rouses them to wild anger, and they abuse the Dominican nuns who come to nurse them. The consciousness of humanity and its responsibilities and dignity must, indeed, be driven out by suffering when a patient strikes his nurse when she is dressing his wounds. This one of these negro lepers did to the sister who was attending him, a refined and gentle Frenchwoman of noble birth. The outrage was, of course, not resented; perhaps it would be better for the patients themselves, as well as for the sisters, if some sterner discipline were introduced. But the Trinidad lepers seem to have few ameliorations of their lot. They are avoided, naturally enough by the inhabitants of the island, and visitors are few and far between. Nor does the charity of the outside world often seek them out. That they are not insusceptible to gratitude may be inferred from one anecdote. A Trinidad gentleman had made friends with one of the lepers, and when he left the island to take up his abode in England he promised, if ever he came back, to come and see him. Neither expected that the promise would ever be kept; but the gentleman revisited Trinidad, and went to the settlement, taking with him a supply of tobacco sufficient to last his leper friend for several months. "The poor wretch fairly danced with joy," says the writer who describes the scene, "and you forgot the hideousness of his appearance and the loathsomeness of the disease in the deep pathos of the scene." Evidently there is something to be made even of the negro leper. To be an outcast on the earth, an object of dread and horror, does not tend to render any man more amiable; and perhaps the fact that the Dominican nuns have renounced the world, though voluntarily, prevents their patients from appreciating the devotion of their lives. Kindness from those who are free to enjoy seems to appeal to them more. It is curious that their greatest delight is a display of fireworks, and doubtless a very simple show of pyrotechnics would content them. Is it not strange that a sufferer from a horrible and fatal disease can be comforted by a few rockets and squibs?

